# HOME AWAY FROM HOME: SOCIODEMOGRAPHIC AND HUMAN CAPITAL PREDICTORS OF NURSING HOME USAGE

**DOI:** 10.1093/geroni/igac059.2421

**Published:** 2022-12-20

**Authors:** Cayley Ryan, Liying Luo

**Affiliations:** Pennsylvania State University, University Park, Pennsylvania, United States; Pennsylvania State University, University Park, Pennsylvania, United States

## Abstract

The U.S. is experiencing a demographic shift characterized by a significant segment of the population entering older age groups and beginning to require specialized eldercare, however little is known about the influence of demographic characteristics on individuals’ involvement with long-term care institutions. I use data from the Health and Retirement Study and logistic regression models to examine how the identities and resources people bring to the later stages of their lives influence their use of nursing homes. I find that individuals’ levels of human capital – measured through their educational attainment – have the strongest influence on their use of nursing homes, with individuals with college degrees having significant increases in the odds of ​reporting any nursing home stay and in the expected count of nursing home stays. I posit multiple pathways by which education could increase the likelihood of using institutionalized care, including through the social mobility of other family members as well as the ability to navigate healthcare resources and evaluate eldercare options. This detailed examination of the intersection between older ages, individual characteristics, the accumulation of different kinds of capital over the life course, and the involvement with formal care facilities provides valuable insight into the relationship between individuals and institutions across older ages and can inform policies attempting to prepare the landscape of formal care institutions for the influx of possible consumers.

